# miR-548ac and miR-378j as Potential Diagnostic Biomarkers in Gastric Cancer: A Case-Control Study

**DOI:** 10.34172/mejdd.2025.434

**Published:** 2025-07-30

**Authors:** Seyed Mahdi Mousavi, Sogand Vahidi, Seyedeh Elham Norollahi, Kosar Babaei, Ebrahim Mirzajani, Pirouz Samidoust, Zivar Salehi, Ali Akbar Samadani

**Affiliations:** ^1^Department of Biology, Faculty of Sciences, University of Guilan, Rasht, Iran; ^2^Medical Biology Research Center, Kermanshah University of Medical Sciences, Kermanshah, Iran; ^3^Cancer Research Center and Department of Immunology, Semnan University of Medical Sciences, Semnan, Iran; ^4^Non-communicable Diseases Research Center, Neyshabur University of Medical Sciences, Neyshabur, Iran; ^5^Department of Biochemistry and Biophysics, School of Medicine, Guilan University of Medical Sciences, Rasht, Iran; ^6^Razi Clinical Research Development Unit, Razi Hospital, Guilan University of Medical Sciences, Rasht, Iran; ^7^Guilan Road Trauma Research Center, Trauma Institute, Guilan University of Medical Sciences, Rasht, Iran

**Keywords:** Gastric cancer, MicroRNAs, miR-548ac, miR-378j, Gene expression, Diagnostic markers, Prognostic indicators

## Abstract

**Background:**

Gastric cancer (GC) is a leading cause of cancer-related deaths worldwide. Despite improvements in diagnostic techniques, GC is often diagnosed at advanced stages. MicroRNAs (miRNAs) have become recognized as important regulators in cancer development, influencing processes such as cell proliferation, migration, invasion, and apoptosis. This study examined the expression levels of two microRNAs, miR-548ac and miR-378j, in GC tissue.

**Methods:**

Tumor and margin tissues were collected from 20 patients diagnosed with gastric adenocarcinoma, and control tissue samples were obtained from 20 healthy individuals. Total RNA was extracted, and cDNA synthesis was performed. Quantitative real-time polymerase chain reaction (qPCR) was used to assess the expression levels of miR-548ac and miR-378j, with miR-16 as the internal control. Statistical analysis was performed using REST software, with SPSS software used for further clinical correlation analysis.

**Results:**

The expression of miR-548ac was significantly upregulated in gastric tumor tissues compared with margin tissues and healthy samples (*P*=0.002). miR-378j expression, on the other hand, showed no significant difference between tumor and normal tissues (*P*=0.05). Statistical analysis revealed a borderline significant association between miR-548ac expression and tumor stage and grade, but found no significant correlation with lymph node or distant metastasis. Additionally, the miR-378j expression did not show significant correlations with any clinicopathological features.

**Conclusion:**

miR-548ac is significantly upregulated in GC tissues and may serve as a potential biomarker for GC diagnosis and prognosis, particularly in more advanced stages. In contrast, miR-378j does not appear to have a strong association with GC and may not be helpful as a diagnostic marker.

## Introduction

 Gastric cancer (GC) is known as one of the most common cancers worldwide. It is the fourth cancer with high prevalence and the second cancer with high mortality.^1^ This cancer is multi-factorial and is caused by the interaction of genetic, epigenetic, and environmental factors, which include *Helicobacter pylori* infection, smoking, high salt consumption, and obesity.^2^ miRNAs play an important role in the occurrence and development of GC. The spread of GC, growth, migration, invasion, apoptosis, and suppression of cancer cells are part of the roles of these small molecules in GC.^3^ The changes in the expression of miRNAs in this cancer, similar to other cancers, are significant, among which increase the expression of miR-17, miR-20a, miR-125b, miR-196a, miR-199a, miR-940 and decrease the expression of miR- 148a, miR-145, miR-133b, miR-1207, miR-1182, miR-506, miR-302 have been noted. Aberrant expression of miRNAs, which leads to their action as oncogenes or tumor suppressors, depending on the expression pattern in different tissues, can be observed in several cancers, including GC. For example, miR-383-5P, miR-411, miR-299-5p, miR-216a, and miR-146a were dysregulated in GC.^4-6^ miR-378 has been reported to play an important role in many cancers. According to the studies, the overexpression of miR-378 prevents the proliferation of colon cancer cells in vitro by inducing apoptosis. It inhibits the migration and invasion of colon cancer cells by inhibiting EMT genes (epithelial-mesenchymal transition). miR-378 by targeting SDAD1 (SDAD1 is a direct target gene of miR-378 that targets the 3’UTR region of SDAD1 to regulate its expression negatively) from cancer cell proliferation, migration, and invasion. Colon prevents and defines miR-378 as a potential target for the diagnosis and treatment of colon cancer.^7^ It was also found that overexpression of miR-378 significantly inhibits the expression of two important transcription factors, TCF-4 and LEF-1. Also, overexpression of miR-378 significantly inhibited the expression of β-catenin and Ki-67. In addition, it was found that miR-378 suppresses the malignant behavior of colon cancer cells by inhibiting the Wnt/β-catenin pathway. During the research, it was found that miR-378 prevents the proliferation of colon cancer cells by inducing apoptosis, and this microRNA plays a tumor suppressor role in colon cancer cells. miR-378 can partially inhibit tumor growth and invasion by targeting vimentin in colorectal cancer. miR-378 inhibits the growth and proliferation of tumor cells in hepatocellular carcinoma^8^ miR-378 has biological functions that regulate various tumor cell processes, including cell proliferation, migration, invasion, and drug resistance. Also, in glioma, decreased miR-378 levels indicate high tumor invasion and poor prognosis.^9^ miR-548 is a large and weak mammalian-specific miRNA gene family consisting of 68 members, and several members of this family have been reported to be associated with cancer. For example, miR-548b-3p acts as a tumor suppressor in lung cancer and as an anti-oncogenic regulator in breast cancer. The results show that miR-548ac is an important tumor suppressor in laryngeal cancer, and suppressing TMEM158 causes apoptosis in laryngeal cancer cells.^10^ miR-548ac suppresses the expression of TMEM158 by directly binding to its 3’-UTR. miR-548ac affects the survival, colony formation, cell cycle distribution, and apoptosis of laryngeal cancer cells. Several pieces of evidence have shown the functional role of miR-548 in various cancers.^11^ The expression and function of miR-548 were investigated in GC. miR-548 was upregulated in GC, which was associated with lymph node metastasis and TNM stage of patients. Patients with relatively high expression of miR-548 had poor survival. miR-548 was identified as a prognostic indicator of GC and also increased its proliferation, migration, and invasion. miR-548 was involved in the progression of GC and predicted the prognosis of patients. Inhibition of miR-548 may be a new therapeutic strategy for GC.^12,13^

 This study aimed to investigate the expression of miR-548ac and miR-378j in gastric adenocarcinoma tissues compared with margin tissues and healthy controls, to identify potential biomarkers for GC diagnosis and progression. While the role of various miRNAs in cancer has been studied, the specific involvement of miR-548ac and miR-378j in GC, especially in the Iranian population where GC is prevalent, is less explored. The research focuses on these miRNAs and their correlation with clinicopathological features.

## Materials and Methods

###  Sample Collection and Preparation

 Twenty patients diagnosed with gastric adenocarcinoma were recruited from the surgery department of Razi hospital. The diagnosis was confirmed via histopathological examination. Tumor tissue and margin tissue (3-7 cm from the tumor margin) were collected during surgery. 20 healthy individuals were recruited from a gastroenterology clinic in Guilan province. These individuals underwent endoscopy for routine check-ups and had no history of gastrointestinal disorders. The status of all tissue was confirmed by histopathological examination. Written informed consent was obtained from all healthy participants. All samples (tumor, margin, and healthy control tissues) were immediately stored at -80 °C.

###  RNA Extraction and Quality Assessment

 Total RNA was extracted from tissue samples using the standard phenol-chloroform method. Briefly, approximately 10-100 mg of tissue was homogenized in liquid nitrogen. The homogenized tissue was lysed in 800 µL of RNSol H solution, followed by the addition of 200 µL of chloroform. The mixture was centrifuged, and the aqueous phase was transferred to a new tube. Isopropanol was added to precipitate RNA, and the RNA pellet was washed with 75% ethanol. The RNA pellet was air-dried and resuspended in DEPC-treated water. RNA quality was assessed by 1.5 % agarose gel electrophoresis and a spectrophotometer, Nanodrop. Finally, it was transferred to a -70-degree freezer for use in the following steps and stored.

###  cDNA Synthesis

 cDNA synthesis was performed using the RevertAid First Strand cDNA Synthesis Kit (Thermo Scientific). Two microliters of total RNA were reverse transcribed in a 20 µL reaction volume containing 5X Reaction Buffer, 10 mM dNTP Mix, 20 U/µL RevertAid Reverse Transcriptase, and a specific primer. The reaction mixture was incubated at 70 °C for 5 minutes, followed by incubation at 25 °C for 5 minutes. Reverse transcription was then carried out at 50 °C for 60 minutes, followed by inactivation at 70 °C for 10 minutes. The synthesized cDNA was stored at -20 °C for further analysis.

###  MicroRNAs Regulation Analysis

 Primers and probes targeting hsa-miR-548ac and hsa-miR-378j were designed based on sequences retrieved from the miRBase database. Bioinformatics tools such as GeneRunner, Oligo7, Oligo Analyzer, and sRNAprimerDB were used to identify optimal primer and probe sequences. Primer specificity was ensured by selecting sequences specific to the target gene. The characteristics and specificity of each primer and probe were evaluated using the aforementioned bioinformatics tools (Table 1).

**Table 1:** Primer sequences of all microRNAs

**microRNAs**	**Primer sequence (FAM-5′ to 3′-TAMRA)**
RT-miR-548ac	GTCGTATCCAGTGCTGCGACCGTATGGATGTGTCTGCGGCGTTTTATCATGCACTGGATACGACCAAAAGT
F-miR-548ac	GTCAGCAAAAACCGGCAATTAC
RT-miR-378j	GTCGTATCCAGTGCTGCGACCGTATGGATGTGTCTGCGGCGTTTTATCATGCACTGGATACGACTTCTGG
F-miR-378j	TGACGACTGGATTTGGAGC
R-Universal	GTATCCAGTGCTGCGACC
Probe-Universal	TGGATGTGTCTGCGGCGTTTTATCAT
RT-miR-16	GTCGTATCCAGTGCAGGGTCCGAGGTATTCGCACTGGATACGACCGCCAA
Forward miR-16	CGCGCTAGCAGCACGTAAAT
Universal Reverse	CCAGTGCAGGGTCCGAGGTA
Probe miR-16	ATACGACCGCCAATAT

 To quantify the expression levels of hsa-miR-548ac and hsa-miR-378j, quantitative real-time PCR (qPCR) was performed using the TaqMan method. The internal control gene, miR-16, was used as a reference gene to normalize gene expression. qPCR reactions were performed in a 20 µL reaction volume containing 12.5 µL of TaqMan Universal PCR Master Mix, 1 µL of forward primer, 1 µL of reverse primer, 0.5 µL of TaqMan probe, 2 µL of cDNA template, and 8 µL of nuclease-free water. Each sample was analyzed in duplicate to minimize experimental variation. The qPCR reactions were performed on a real-time PCR system with the following thermal cycling conditions: Initial denaturation at 95 °C for 10 minutes, denaturation at 95 °C for 15 seconds, and annealing and extension at 60 °C for 1 minute, carried out for 40 cycles. Finally, the relative expression levels of target miRNAs were calculated using the 2^-ΔΔCt^ method. Statistical analysis was performed using REST software.

###  Bioinformatics Analysis of miR-548ac and miR-378j Target Genes

 We utilized several bioinformatics databases to predict the target genes of miR-548ac and miR-378j. These databases included TargetScan, miRWalk, miRTarBase, miRTar2GO, miRDB, miRSystem, DIANA, miRanda, miRBridge, and RNA22. We systematically analyzed the predicted target genes from each database and identified overlapping targets between the two microRNAs. The resulting network interactions were visualized, demonstrating the relationships between the miRNAs and their respective target genes. The green nodes in the diagram represent the common target genes shared by miR-548ac and miR-378j, highlighting potential synergistic regulatory mechanisms.

###  Comprehensive Analysis of miRNA-Target Interactions in Gastric Cancer Using the miRPath Database

 To investigate the regulatory role of miRNAs in GC, we utilized the miRPath v4.0 database.^14^ A set of miRNAs was analyzed for their target interactions, including hsa-miR-378j, hsa-miR-548ac, and three other known GC-related miRNAs: hsa-miR-1260b, hsa-miR-320a-3p, and hsa-miR-320a-5p, in the Kyoto Encyclopedia of Genes and Genomes (KEGG) GC pathway. Additionally, we utilized the “clusterProfiler” package from the Bioconductor software suite in R for our Gene Ontology (GO) analysis and used the miRPath v4.0 database to visualize the relationship between miRNAs and enriched pathways. This analysis categorized the five miRNAs according to their biological processes (BP), cellular components (CC), and molecular functions (MF), providing a clearer understanding of their roles within the cell. Statistical significance was determined using a classical enrichment analysis approach with a false discovery rate (FDR) and *P* value correction threshold of 0.05.

###  Statistical Analysis

 The REST software was utilized to analyze the data obtained from real-time PCR. The data were further analyzed using SPSS software, version 22. T-test, ANOVA, and regression analysis were employed to evaluate the expression of the studied genes. The fold change method was used to compare the expression levels of these genes with those in healthy samples. The changes in gene expression were normalized using an internal control gene, and the expression levels in the control or healthy samples were then examined. Values with a significance of *P* < 0.05 were considered statistically significant.

## Results

 Patients’ samples were analyzed and categorized based on sex, age, tumor stage, tumor grade, distant metastasis, and lymph node metastasis. Additionally, the expression levels of miR-378j and miR-548ac were evaluated, with statistical significance (*P* values) calculated for each characteristic to assess potential associations with these microRNAs. According toTable 2, sex shows a statistically significant association with miR-378j expression (*P* = 0.04). miR-548ac is close to significant with respect to sex (*P* = 0.05) and tumor stage (*P* = 0.08). Other characteristics, such as age, grade, liver metastasis, and distant metastasis, did not show statistically significant associations with either miR-378j or miR-548ac. These results suggest that sex and possibly tumor stage may be relevant to miRNA expression in this patient population.

**Table 2:** Characteristics of patients, along with their clinical and pathological features

**Characteristics**	**Total Patients (N=20) (%)**	**↓ miR-378j ** ***P*** ** value**	**↓miR-548ac ** ***P*** ** value**
Sex		0.04	0.05
Female	8 (40)		
Male	12 (60)		
Age		0.03	0.06
< 60 years	10 (50)		
≥ 60 years	10 (50)		
Stage		0.3	0.08
I	0 (0)		
II	3 (15)		
III	9 (45)		
IV	8 (40)		
Grade		0.5	0.08
Well-differentiated	1 (5)		
Moderate differentiate	5 (25)		
Poorly differentiate	11 (55)		
Undifferentiated	3 (15)		
LM		0.5	0.7
Yes	16 (80)		
No	4 (20)		
DM		0.8	0.7
Yes	11 (55)		
No	9 (45)		

###  Analysis of miR-378j and miR-548ac Expression in Patients with Gastric Cancer

**Figure 1:**
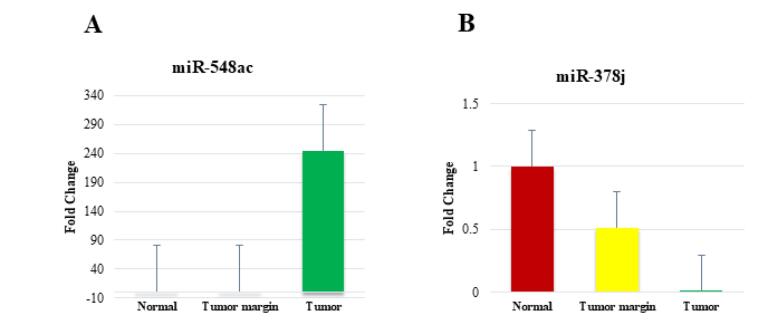
Analysis of the scatter plot for miR-378j and miR-548ac highlights that miR-378j is significantly down-regulated in tumoral tissues compared with non-tumoral tissues across the majority of patients with gastric cancer. The relatively high R^2^ value (0.5676) further supports this trend, indicating a strong, consistent pattern of down-regulation

 Real-time PCR reaction was performed using cDNA synthesized from the tumor, tumor margin, and control samples for mir-548ac and mir-378j. The expression level in the tumor tissue is significantly higher compared with both the normal and tumor margin tissues. The fold change of miR-548ac in the tumor tissue is approximately 250, which is a very substantial upregulation compared with the normal tissue (*P* = 0.002) (Figure 1A). This suggests that miR-548ac may play an important role in the tumor tissue and could potentially be used as a biomarker or therapeutic target.

 The expression level of miR-378j in the normal tissue is higher compared with the tumor margin and tumor tissues. The fold change of miR-378j in the tumor tissue is around 1, indicating no significant difference in expression compared with the normal tissue (*P* = 0.05) (Figure 1B).

 This suggests that miR-378j may not be strongly associated with tumor tissue and may not be a reliable biomarker for this particular cancer type. In summary, the data indicate that miR-548ac is significantly upregulated in tumor tissue, whereas miR-378j does not exhibit a substantial change in expression. These findings suggest that miR-548ac could be a more promising biomarker or therapeutic target compared with miR-378j for the cancer type represented in these samples.

**Figure 2:**
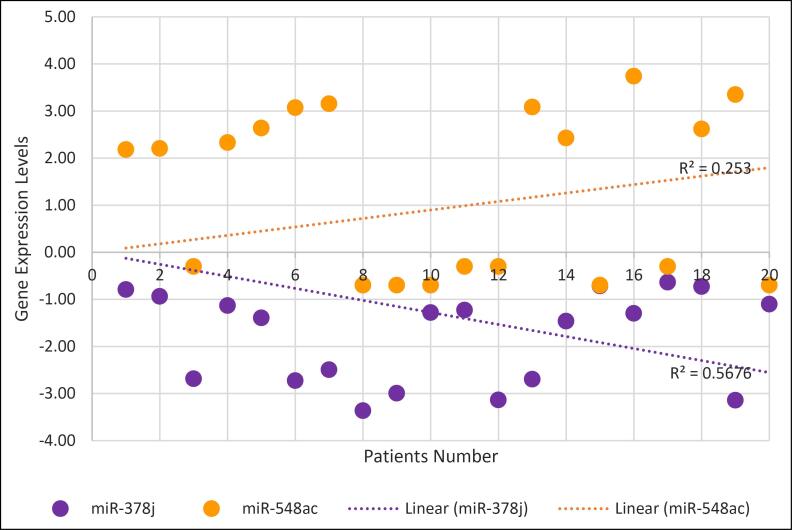
Scatter plot analysis of relative expression of miR-378j and miR-548ac in patients with gastric cancer. The Y axis indicates the logarithm of relative gene expression. The upper part of the graph indicates up-regulation in the tumoral compared with the non-tumoral tissue; the lower part of the graph indicates down-regulation in the tumoral compared with the non-tumoral tissue

 miR-548ac, on the other hand, shows a general trend of up-regulation in tumor tissues, but the variability in its expression is more pronounced, as suggested by the lower R^2^ value (0.253). This suggests that while miR-548ac may play a role in tumor biology, its expression levels are less predictable across different patients (Figure 2).

###  The Association of miR-548ac and miR-378j Expression with Clinicopathological Qualifications

**Figure 3:**
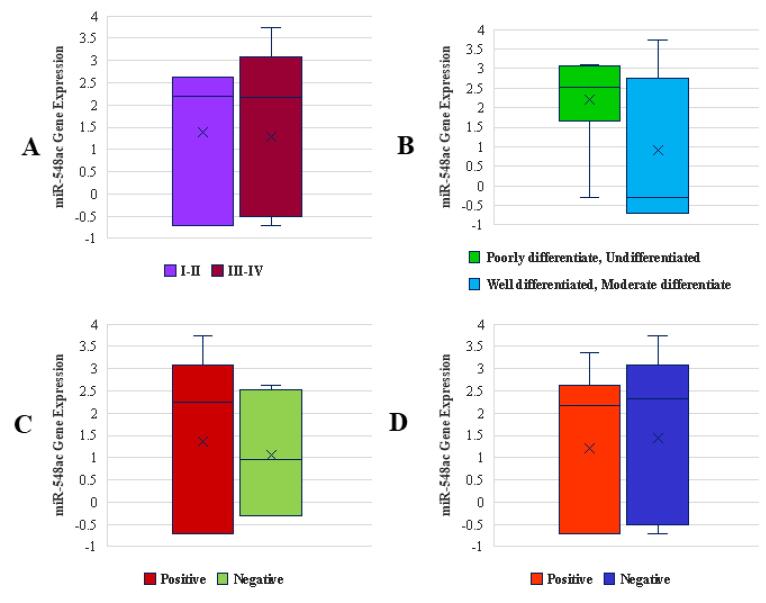
The association between miR-548ac gene expression and various clinicopathological characteristics in gastric cancer patients, based on the Chi-squared test. A. tumor stage (Stage I-II vs. Stage III-IV), B. tumor grade (Poorly differentiated/Undifferentiated vs. Well differentiated/Moderately differentiated), C. lymph node metastasis (Positive vs. Negative) and D. distant metastasis (Positive vs. Negative)

 miR-548ac expression shows a borderline significant association with both tumor stage and tumor grade (*P =*0.08 for both) (Figure 3A, 3B). This suggests that miR-548ac may play a role in the progression of GC, particularly in more advanced and poorly differentiated tumors. However, no significant association was observed between miR-548ac expression and either lymph node metastasis (Figure 3C) or distant metastasis (Figure 3D) (both *P* = 0.7). This suggests that miR-548ac expression is not directly related to metastatic spread, either to lymph nodes or distant organs.

**Figure 4:**
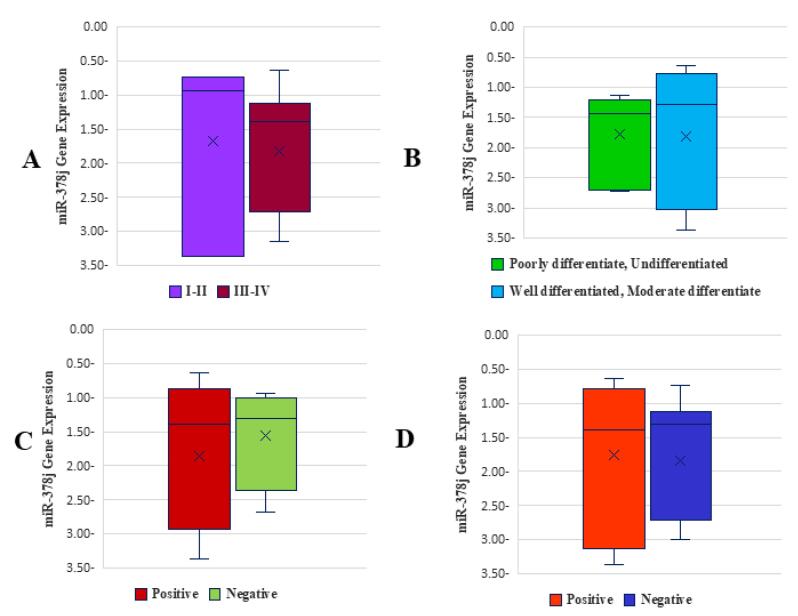
The association between miR-378j gene expression and various clinicopathological characteristics in gastric cancer patients, based on the Chi-squared test. A. tumor stage (Stage I-II vs. Stage III-IV), B. tumor grade (Poorly differentiated/Undifferentiated vs. Well differentiated/Moderately differentiated), C. lymph node metastasis (Positive vs. Negative) and D. distant metastasis (Positive vs. Negative)

 The expression of miR-378j does not show statistically significant associations with any of the clinicopathological features examined, including tumor stage, tumor grade, lymph node metastasis, and distant metastasis (Figure 4).

 The lack of significant differences in miR-378j expression across these clinical parameters (all *P* > 0.05) suggests that miR-378j may not play a critical role in the progression, aggressiveness, or metastatic behavior of GC in these patients.

###  The Correlation Analysis between miR-548ac and miR-378j Regulation

**Figure 5:**
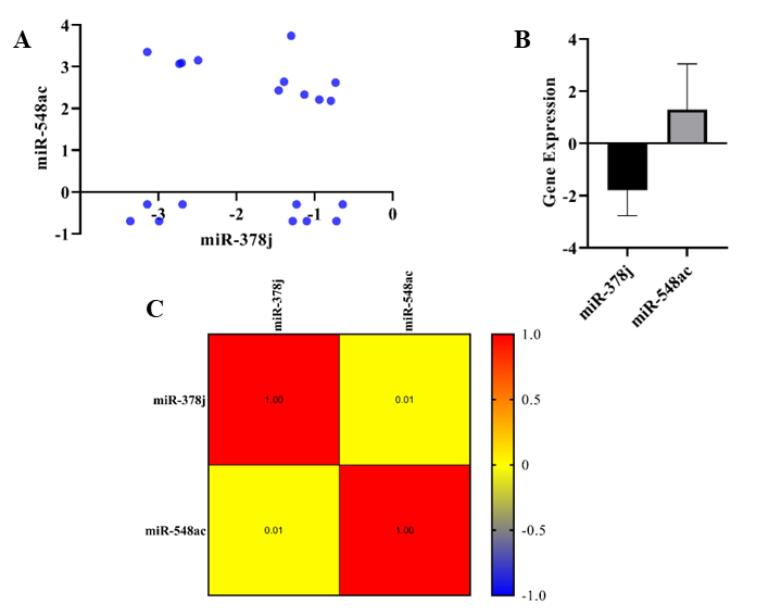
The correlation between the expression of miR-548ac and miR-378j in gastric cancer tumor samples. A. scatter plots, B. bar chart, and C. heatmap

 The results of a Pearson correlation analysis between the expression levels of miR-548ac and miR-378j in GC tumor samples are indicated in Figure 5.

 The scatter plots (Figure 5A) depict the relationship between the upregulation of miR-548ac and the downregulation of miR-378j across different tumor samples in patients with GC. The points in the scatter plots are widely spread without any clear linear relationship. There is no discernible pattern suggesting either a positive or negative correlation between the two variables. The Pearson correlation coefficient (*r* = 0.01) indicates an almost zero correlation between the expression levels of these two miRNAs. This implies that changes in miR-548ac expression do not correspond to changes in miR-378j expression. The *P* = 0.96 is not statistically significant, reinforcing that there is no meaningful correlation between miR-548ac and miR-378j in this dataset.

 The bar graph (Figure 5B) also visualizes the statistical result of the Pearson test, showing the correlation coefficient (*r* = 0.01) between miR-548ac and miR-378j expression.

 Accordingly, the heatmap (Figure 5C) visually represents the correlation matrix between miR-548ac and miR-378j. Each cell in the heatmap shows the Pearson correlation coefficient between pairs of variables (miR-548ac and miR-378j). The diagonal cells (in red) represent the self-correlation of each miRNA, which is always 1.00 (perfect correlation with itself). The off-diagonal cells indicate the correlation between miR-548ac and miR-378j, which is 0.01 in both directions, confirming the absence of a significant correlation. The color scale bar on the right indicates that the yellow color corresponds to a correlation value near zero, further visualizing the weak correlation.

###  Interaction Network of miR-548ac and miR-378j Target Genes

**Figure 6:**
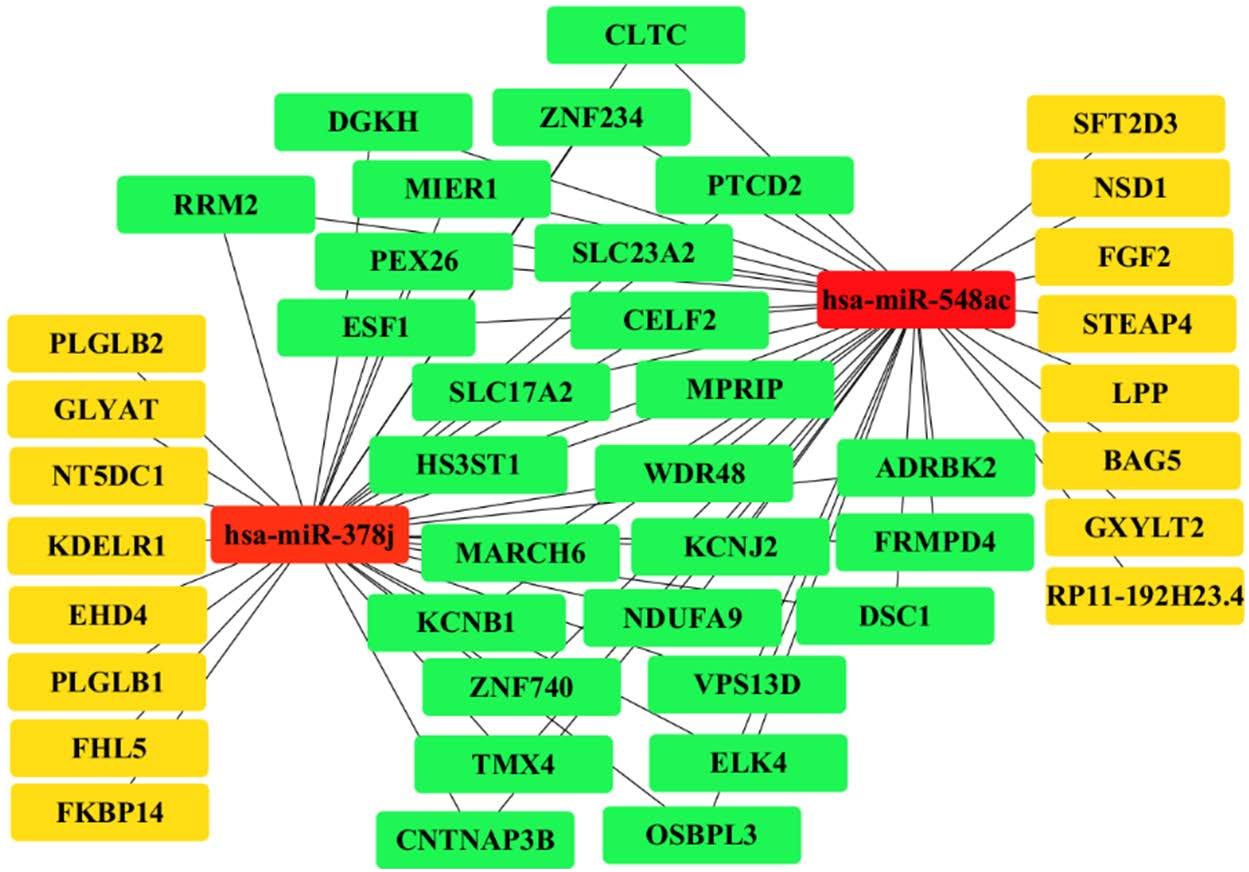
Theinteraction network illustrates the network of target genes predicted for miR-548ac and miR-378j. Green nodes represent the common target genes shared by both microRNAs, while yellow nodes indicate unique targets for each miRNA. The connecting lines represent the interactions between microRNAs and their respective target genes, indicating potential regulatory relationships

 The network analysis of target genes for miR-548ac and miR-378j revealed a complex interaction landscape. Among the common target genes, several top nodes stood out due to their central roles in various biological pathways. We identified a set of overlapping target genes, represented as green nodes in the interaction diagram (Figure 6). This visualization highlights the key relationships between the two microRNAs and their shared targets, indicating potential collaborative regulatory mechanisms. These pathways are known to be involved in critical cellular processes such as growth, apoptosis, and signal transduction. The prominence of these genes in the network suggests that miR-548ac and miR-378j may collectively influence the expression of these genes, thereby affecting cellular functions and potentially contributing to disease processes.

 This analysis underscores the significance of miRNA interactions in gene regulation and highlights the need for further investigation into the specific pathways involved. Including these top nodes also provides a foundation for future studies aimed at understanding the molecular mechanisms underlying miRNA-mediated regulation.

###  GO and KEGG Pathway Analysis 

**Figure 7:**
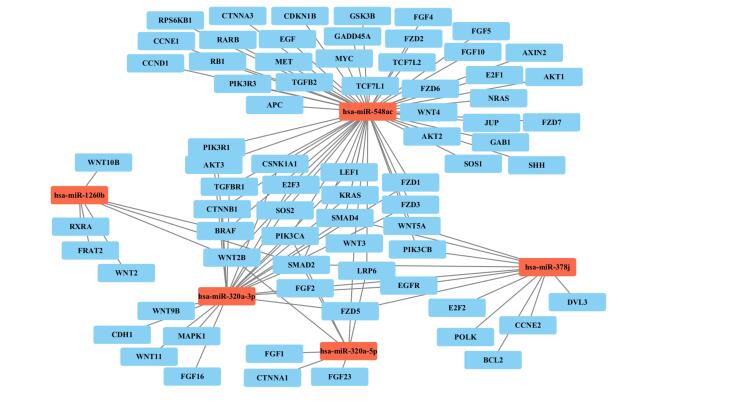
The miRNA-mRNA regulatory network of hsa-miR-378j, hsa-miR-548ac, hsa-miR-1260b, hsa-miR-320a-3p, and hsa-miR-320a-5p and the targeted genes associated with gastric cancer

 The analysis from the miRPath database highlights significant connections between these miRNAs and GC, examining their potential roles in regulating key genes involved in the disease’s pathogenesis (Figure 7). Our analysis identified 73 target genes associated with GC that were significantly regulated by hsa-miR-378j, hsa-miR-548ac, hsa-miR-1260b, hsa-miR-320a-3p, and hsa-miR-320a-5p (*P* = 0.0000110, FDR = 0.000179). The enriched genes included critical oncogenic drivers such as SMAD2, WNT2, BRAF, and PIK3CA. Notably, hsa-miR-1260b targets several critical genes, including SMAD2, FRAT2, WNT2, RXRA, WNT2B, BRAF, and WNT10B, and plays a vital role in regulating pathways associated with cell migration and invasion, which are crucial for cancer metastasis. Similarly, both hsa-miR-320a-3p and hsa-miR-320a-5p target genes such as SMAD2, E2F3, SOS2, LEF1, MAPK1, PIK3CA, and CTNNB1, highlighting their involvement in regulating the cell cycle and apoptosis, thereby indicating their potential role in tumorigenesis. Furthermore, hsa-miR-378j targets genes like SMAD2, EGFR, SMAD4, BCL2, and FGF2, contributing to enhanced cell survival and proliferation, which can significantly impact cancer growth. Lastly, hsa-miR-548ac, by targeting SMAD2, KRAS, PIK3CA, and BRAF, may influence oncogenic pathways, thereby playing a crucial role in the progression of GC.

 Several common target genes were identified among the miRNAs, including SMAD2, EGFR, PIK3CA, BRAF, and WNT2B. The presence of these common genes suggests a shared regulatory mechanism through which these miRNAs may influence GC development. This overlap suggests that these miRNAs may be part of a coordinated network influencing critical pathways, including Wnt signaling, TGF-β signaling, and cell survival pathways.

**Figure 8:**
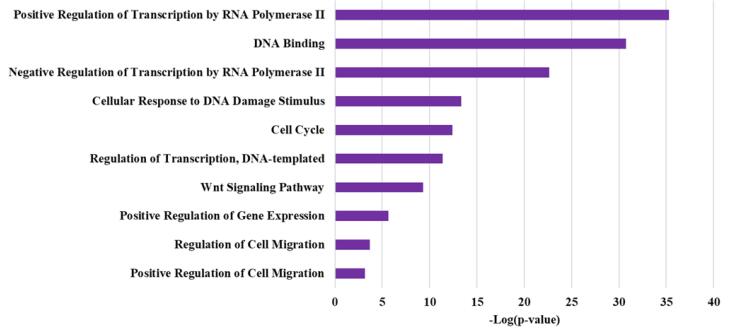
GO enrichment analysis of hsa-miR-378j, hsa-miR-548ac, hsa-miR-1260b, hsa-miR-320a-3p, and hsa-miR-320a-5p predicted by microT-CDS

 Furthermore, GO analysis revealed enrichment for specific GO categories associated with these miRNAs. This analysis identified several key signaling pathways that are regulated by each of the five microRNAs hsa-miR-378j, hsa-miR-548ac, hsa-miR-1260b, hsa-miR-320a-3p, and hsa-miR-320a-5p, which significantly affect GC, as indicated by their low *P* values and FDR. In Figure 8, there are ten signaling pathways with *P* values and FDRs below 0.001, highlighting their critical roles in cancer progression. Notably, the Positive Regulation of Transcription by RNA Polymerase II pathway exhibited the most substantial influence, with a *P* value of 5.47e-36 and an FDR of 2.05e-32, suggesting its critical role in gene expression modulation during cancer progression. Additionally, both the Negative Regulation of Transcription by RNA Polymerase II and Regulation of Transcription, DNA-templated pathways also demonstrated significant effects, with *P* values of 2.20e-23 and 9.73e-26, respectively. The Cell Cycle pathway, essential for cellular proliferation, showed a *P* value of 4.03e-13, indicating its relevance in tumor growth. Furthermore, the Wnt Signaling Pathway was significantly associated with GC, with a *P* value of 4.68e-10, highlighting its involvement in cell migration and differentiation. Lastly, the Positive Regulation of Cell Migration pathway, crucial for metastasis, had a *P* value of 6.97e-07, reinforcing the importance of migration-related processes in GC pathology. These findings underscore the complex signaling mechanisms that contribute to GC development and progression.

## Discussion

 GC remains a major global health challenge, being one of the leading causes of cancer-related mortality worldwide. Despite recent advances in diagnostic tools and therapeutic strategies, GC is often diagnosed at advanced stages, significantly limiting treatment options and reducing survival rates.^15^ As a result, the need for sensitive and specific biomarkers for early diagnosis and prognosis is critical. miRNAs have emerged as promising candidates for cancer biomarkers due to their stability in biological samples and their regulatory roles in various cellular processes, including proliferation, migration, invasion, and apoptosis.^16^ In this study, we focused on two specific miRNAs, miR-548ac and miR-378j, to evaluate their potential as biomarkers for GC. Our findings demonstrate a significant upregulation of miR-548ac in gastric tumor tissues compared with both tumor margin tissues and healthy controls. The observed fold change of approximately 250 in tumor tissues underscores the substantial involvement of miR-548ac in gastric tumorigenesis. The up-regulation of miR-548ac suggests that it may function as an onco-miR (oncogenic miRNA), promoting tumor growth or survival. However, the variability in its expression pattern indicates that its role may not be uniform across all patients or may be influenced by additional factors. These results are consistent with findings from previous studies on miR-548ac in other types of cancers. For example, a study by Nolte and colleagues demonstrated that miR-548ac was upregulated in kidney cancer cells in response to apoptosis inducers, suggesting its role in promoting tumor cell survival.^17^ Similarly, miR-548ac has been identified as a key miRNA involved in breast cancer, further supporting the oncogenic potential of this miRNA across various tumor types.^18,19^ Although these studies were conducted in cancers other than GC, they align with our findings that miR-548ac may act as an onco-miR, contributing to tumor growth and progression. Moreover, we found a borderline significant association between miR-548ac expression and tumor stage and grade. This suggests that miR-548ac expression levels may increase as the tumor progresses and becomes more aggressive. Similar findings have been reported in other cancer types. For example, Hecker et al^20^ observed that miR-548ac expression correlated with disease progression in multiple sclerosis, while Hui et al^21^ found increased miR-548ac expression in adolescent idiopathic scoliosis, indicating that miR-548ac may be involved in various pathological processes beyond cancer. In the context of GC, our study suggests that miR-548ac could serve as a valuable biomarker for identifying more advanced and aggressive tumors, similar to its proposed role in other cancers. Despite its upregulation in tumor tissues, miR-548ac did not significantly correlate with lymph node metastasis or distant metastasis. This lack of correlation with metastatic spread contrasts with other studies where miR-548ac was implicated in promoting metastasis. For example, Dahiya and colleagues demonstrated that miR-548ac played a role in regulating platelet mitochondrial function, which could influence cancer cell migration and invasion.^22^ However, in our study, miR-548ac appears to be more involved in tumor development and progression rather than in metastasis. This observation highlights the context-dependent functions of miRNAs, where a miRNA may serve different roles across various cancer types or stages. In contrast to miR-548ac, miR-378j did not show significant differences in expression between tumor tissues, tumor margins, and healthy controls in our study. Furthermore, miR-378j expression was not significantly associated with any clinicopathological features, including tumor stage, grade, lymph node metastasis, or distant metastasis. These findings suggest that miR-378j may not play a critical role in GC, at least in the patient cohort we examined. This result is somewhat surprising given the reported roles of miR-378 family members in other cancers. For example, Berillo et al identified miR-378j as a miRNA involved in colorectal cancer by interacting with the 3’ UTR of the GRK5 gene, which encodes a protein involved in tumorigenesis.^23^ Similarly, miR-378j suppressed liver fibrosis by inhibiting hepatic stellate cells.^24^ In our study, however, the lack of significant findings for miR-378j in GC suggests that its role in this cancer type may be minimal, or that it may function in a context-dependent manner, as seen in other conditions such as liver fibrosis. A potential explanation for these discrepancies could be the tissue-specific expression and function of miRNAs. While miR-378j appears to play significant roles in colorectal cancer and liver fibrosis, its involvement in GC may be limited. This highlights the importance of studying miRNAs in a cancer-specific context. In the case of GC, our data suggest that miR-378j may not be a reliable biomarker for diagnosis or prognosis, contrasting with its more prominent role in other diseases. Interestingly, our correlation analysis between miR-548ac and miR-378j expression in GC tissues revealed no significant relationship, suggesting that these two miRNAs likely operate independently within the molecular pathology of GC. This lack of correlation further supports the idea that miR-548ac and miR-378j may have distinct roles in tumor biology. While miR-548ac appears to act as an oncogenic miRNA, promoting tumor development, miR-378j may not be as relevant in this context. These findings are in contrast to studies in other cancers where miR-378j has been shown to interact with other miRNAs or genes involved in cancer progression. Dix and colleagues identified novel interactions between miR-378j and other miRNAs in dendritic cells during fungal infections, suggesting that miR-378j may function within a complex regulatory network.^25^ However, in GC, our results suggest that miR-548ac and miR-378j likely operate through distinct, non-overlapping pathways, further underscoring the tissue- and cancer-specific roles of these miRNAs. The potential of miR-548ac as a biomarker for GC is further strengthened by its significant upregulation in tumoral tissues and its association with tumor stage and grade. This suggests that miR-548ac could potentially serve as a diagnostic and prognostic marker, especially for identifying more advanced and aggressive forms of GC. However, the absence of a significant relationship between miR-548ac and metastasis indicates that its utility may be more limited to tumor detection and progression rather than predicting metastatic potential. The bioinformatics analysis identified specific miRNAs linked to GC, particularly hsa-miR-1260b, hsa-miR-320a-3p, hsa-miR-320a-5p, hsa-miR-378j, and hsa-miR-548ac, highlighting their potential roles in the disease’s pathogenesis. Each of these miRNAs targets critical genes involved in essential biological processes, including cell migration, invasion, cell cycle regulation, and apoptosis. The overlapping targets, such as SMAD2, PIK3CA, and BRAF, suggest that these miRNAs may operate within shared regulatory networks, influencing tumor progression and metastasis. Notably, hsa-miR-1260b and hsa-miR-378j enhance cell survival and proliferation, which are fundamental hallmarks of cancer. The findings highlight the importance of these miRNAs not only as potential biomarkers for GC diagnosis but also as therapeutic targets for intervention strategies aimed at modulating their regulatory effects on oncogenic pathways. Further studies are needed to elucidate the precise mechanisms through which these miRNAs influence GC biology and to explore their clinical relevance in treatment outcomes.

 Accordingly, combining multiple miRNAs into a diagnostic panel has the potential to significantly enhance both the sensitivity and specificity for GC diagnosis. Individual miRNAs often exhibit variable diagnostic performance; however, a panel approach captures the biological complexity of GC, which is characterized by heterogeneous molecular alterations. By integrating several miRNAs, we can leverage their synergistic effects, allowing for improved detection of cancerous tissues and enhancing sensitivity. This multi-miRNA strategy also reduces the likelihood of false positives and negatives, thereby increasing specificity.^26,27^ Our analysis indicates significant associations between specific miRNAs and GC, supported by low P values and FDRs, suggesting that their combined expression profiles can refine diagnostic accuracy. Furthermore, a multi-miRNA panel is likely to be more clinically relevant, providing a robust tool for early detection and monitoring, ultimately leading to better patient outcomes. Thus, the integration of multiple miRNAs into a diagnostic framework holds promise for improving GC diagnosis, and further validation in larger cohorts will be essential to confirm these findings and establish clinical utility.

 While our study provides valuable insights into the roles of miR-548ac and miR-378j in GC, several limitations should be acknowledged. First, the relatively small sample size (20 patients) may limit the generalizability of our findings. Larger studies with more diverse patient populations are needed to validate the expression patterns of miR-548ac and miR-378j in GC. Second, while we identified significant upregulation of miR-548ac, we did not investigate its direct targets or the molecular mechanisms through which it promotes tumorigenesis. Future studies should focus on elucidating the signaling pathways regulated by miR-548ac in GC, as well as exploring its potential as a therapeutic target through miRNA-based interventions. Additionally, the absence of an independent validation cohort limits the reproducibility of our findings, highlighting the need for future studies to use external datasets for validation. Another intriguing aspect of our findings is the potential therapeutic utility of miR-548ac. Given its significant upregulation in tumor tissues, miR-548ac could be explored as a target for miRNA-based therapies, such as miRNA inhibitors. Inhibiting miR-548ac might help suppress tumor growth or enhance the efficacy of existing treatment modalities. However, this approach would require extensive validation in preclinical models before being considered for clinical applications.

## Conclusion

 The findings indicate that miR-548ac is significantly upregulated in tumor tissues compared with both normal tissues and healthy controls, suggesting its involvement in tumor development and progression. Moreover, its expression is borderline and significantly associated with tumor stage and grade, indicating that miR-548ac could be a valuable biomarker in the diagnosis and prognosis of advanced GC. In contrast, miR-378j did not show significant changes in expression or associations with clinicopathological parameters, suggesting it may be less relevant in the context of GC. The lack of correlation between miR-548ac and miR-378j expression further supports the idea that these miRNAs may play distinct roles in the molecular pathology of GC. The results suggest that miR-548ac could be explored as a therapeutic target, as its inhibition may hold promise for limiting tumor progression. Consequently, miR-548ac represents a promising candidate for further investigation as a diagnostic and prognostic biomarker in GC, while miR-378j appears to have a minor role in this disease. Future studies should focus on validating these results in larger patient cohorts and exploring the molecular mechanisms through which miR-548ac contributes to gastric tumorigenesis.
